# The Efficacy and Mid-term Durability of Urethral Sphincter Injections of Platelet-Rich Plasma in Treatment of Female Stress Urinary Incontinence

**DOI:** 10.3389/fphar.2022.847520

**Published:** 2022-02-08

**Authors:** Ching-Hsiang Chiang, Hann-Chorng Kuo

**Affiliations:** Department of Urology, Hualien Tzu Chi Hospital, Buddhist Tzu Chi Medical Foundation and Tzu Chi University, Hualien, Taiwan

**Keywords:** female, stress urinary incontinence, patient reported outcome measures, urethra, urodynamics

## Abstract

**Aims:** This study investigated the therapeutic effect of repeated urethral sphincter injections of autologous platelet-rich plasma (PRP) in treatment of stress urinary incontinence (SUI) in women due to intrinsic sphincter deficiency (ISD) refractory to medical treatment or after the first anti-incontinence surgery.

**Methods:** Twenty-six women with SUI due to urodynamically proven ISD were prospectively enrolled. Five milliliters of PRP (2.5–5 times of the platelet concentrations in peripheral blood) were injected into the external sphincter at 5 sites, with 4 treatments at monthly interval. The primary end-point was post-treatment Global Response Assessment (GRA, scored 0–3) score after four PRP treatments. A GRA ≥ 2 was considered as a successful result. The secondary endpoints included changes in visual analog scale (VAS) of SUI and urodynamic parameters. The follow-up date was 12 months after the fourth PRP treatment.

**Results:** The mean age was 61.7 ± 15.3 years. The overall success rate was 50% with the post-treatment mean GRA of 1.5 ± 1.1. Complete dryness was achieved in 12 patients (46.2%) after the PRP treatment, and 7 (26.9%) kept total continence at 12 months. The mean VAS of SUI score decreased significantly from 6.4 ± 2.3 to 3.9 ± 2.3 after treatment (*p* < 0.001). The abdominal leak point pressure increased significantly from 117.5 ± 63.8 to 133.6 ± 61.7 cmH_2_O (*p* = 0.045). No perioperative adverse events or severe complications occurred, except 1 (3.8%) patient reported straining to void which was self-limited.

**Conclusion:** Repeated urethral sphincter injections of autologous PRP are a safe procedure that provides significant reduction in the severity of female SUI and a mid-term durability, suggesting PRP treatment is effective to increase urethral sphincter resistance for female SUI.

## 1 Introduction

Stress urinary incontinence (SUI), defined as involuntary leakage of urine on effort or exertion, is common in women (Abrams et al., 2002; Agarwal and Agarwal, 2017). Female SUI typically occurs with aging, multiple child births and menopause, and it may impairs the quality of life of the afflicted individual owing to shame and embarrassment caused by incontinence.3.

First-line conservative therapies to deal with female SUI include behavioral modifications, pad use, pharmacotherapy, and pelvic floor exercises (Lukacz et al., 2017; Dumoulin et al., 2018). Once initial conservative treatment fails, surgical interventions such as midurethral slings, bulking agents, and colposuspension to restore urethral competence have been recommended (Gomelsky et al., 2019). These procedures have been widely performed with acceptable cure rate and durability (Freites et al., 2019; Liu and Kuo, 2019). However, patients with mild SUI might hesitate to undergo surgery, especially in the era that several suburethral sling complications have been warned by the FDA. Therefore, less invasive alternatives such as injection therapy with urethral bulking agent or stem cell formulation have been developed (Kirchin et al., 2017). A simple, effective and less invasive management is warranted for this distressing disorder in women with mild to moderate SUI.

Platelet-rich plasma (PRP) is an autologous blood-derived product obtained directly from patients’ own peripheral blood. It comprises a high concentration of platelets and a pool of cytokines, chemokines, and growth factors and is known to contribute to tissue regeneration (Nurden, 2011; Mussano et al., 2016). It is growing in popularity as a therapy to augment wound healing and hasten recovery after muscle and joint injuries, neuropathies, and surgical repair (Mussano et al., 2016; Sánchez et al., 2017). Preliminary studies have shown that repeated PRP injections into the suburothelium are safe and may improve bladder pain and urinary frequency in interstitial cystitis/bladder pain syndrome (Jhang et al., 2019a; Jhang et al., 2019b). As a biological material extracted from the patient’s blood, it is easily produced, relatively inexpensive, and lower potential adverse effects such as foreign body reaction than synthetic materials (Jiang et al., 2021).

According to the potential regenerative effect, PRP injection might benefit the patient with SUI by increasing the sphincter muscle cells as well as the urethral resistance. The concept was first reported in 2016 and has been proved in both animal model and clinical use (Nikolopoulos et al., 2016; Nikolopoulos et al., 2019; Lee et al., 2021). In this study, we performed a clinical trial to investigate the therapeutic efficacy, and durability of autologous PRP urethral sphincter injections for treatment of SUI due to Intrinsic sphincter deficiency in women.

## 2 Methods

This is a prospective, single-center study conducted from April 2018 to October 2020. The study was approved by the Institutional Review Board and Ethics Committee of the Hualien Tzu Chi Hospital (IRB: 107-231-A). Patients were informed about the study rationale and procedures. A written informed consent was obtained from all patients prior to enrollment and treatment. Women who had SUI with videourodynamic study (VUDS) proven ISD were enrolled in this trial. The diagnosis of ISD was made by the findings of urinary leakage in association with urethral incompetence without bladder hypermobility during abdominal straining. All these patients needed pad protection and were refractory to conservative treatment (e.g., lifestyle modifications, pelvic floor muscle training, bladder retraining, and oral pharmacotherapy) or had SUI recurrence after previous surgical procedure by suburethal sling at least 1 year ago. All cases were invastigated thoroughly at enrollment and were excluded if they did not satisfy the inclusion criteria (Supplementary S1). All the enrolled patient had undergone VUDS and abdominal leak point pressure (ALPP) study to prove the diagnosis of ISD. The comprehensive data of age, duration of SUI, medical comorbidities, follow-up months after PRP treatment, and 3-day voiding diary for daily urinary leakage episodes were collected. The severity of SUI was measured by the patient’s self-assessment using a 10-point VAS scale (0: no incontinence, 10: complete incontinence, Table 1), which was newly designed scoring system modified from the Stamey SUI grading system to discriminate SUI severity in more detail (Stamey, 1980). The SUI VAS scale had been validated in our previous study (Jiang et al., 2021). Sequential questionnaires including Urogenital Distress Inventory (UDI-6) and Incontinence Impact Questionnaire (IIQ-7) were also obtained at the outpatient clinic as subjective assessment (Abrams et al., 2003).

**TABLE 1:** Visual analogue scale (VAS) for the assessment of stress urinary incontinence (SUI).

VAS of SUI	Corresponding Stamey grade of SUI	Situation of SUI	Frequency of SUI (on situation)	Pad protection
0	0	Complete dryness	0	No
1	1	Any mild or severe straining situation	1 episode per days to weeks	No/Yes
2	1	Heavy straining/squatting	1 episode per day	No/Yes
3	1–2	Coughing/sneezing/laughing/nighttime/change position	1 episode per day	No/Yes
4	1	Heavy straining/squatting	>1 episode per day	No/Yes
5	1–2	Coughing/sneezing/laughing/nighttime/change position	>1 episode per day	No/Yes
6	2	Walking	<50% situation (every day) & > 1 episode per day	Yes
7	2	Very mild movement/change position	<50% situation (every day) & > 1 episode per day	Yes
8	2	Walking	≥50% situation (every day) & > 1 episode per day	Yes
9	2	Very mild movement/change position	≥50% situation (every day) & > 1 episode per day	Yes
10	3	Any condition; persistent	All the time	Yes

VUDS, ALPP measurements and urethral pressure profilometry (UPP) were routinely performed at baseline and 3 months after fourth PRP treatment. The following VUDS parameters were measured, including maximum flow rate (Qmax), voided volume, post-void residual volume, cystometric bladder capacity, corrected Qmax (Qmax/voided volume^1/2^) and voiding detrusor pressure at Qmax. ALPP measurement was performed at full bladder and the ALPP data was collected by the smallest pressure that patient leaked during several coughs and abdominal straining maneuvers. If there was no leak in response to coughing or Valsalva maneuver, the highest abdominal pressure the patient generated was used to represent the ALPP. The change of the ALPP between baseline and post-treatment was considered as the increment of urethral resistance after PRP treatment. UPP parameters included maximum urethral closure pressure and functional profile length. The description and terminology of the urodynamic parameters were in accordance with the recommendations of the International Continence Society (Abrams et al., 2003).

All eligible patients were admitted for PRP injections. In the morning of the treatment day, a total of 50 ml of peripheral blood was withdrawn, followed by two centrifugation steps in a licensed laboratory for preparation of PRP. A soft spin at 200×g for 20 min at 20°C was initially performed to separate the plasma and erythrocyte layers. The upper plasma layer was aseptically collected and subjected to a second hard spin at 2000×g for 20 min at 20°C. There was in total 5 ml of PRP (approximately 2.5–5 times increase of the platelet concentrations than that in the peripheral blood) for each injection.

**FIGURE 1:**
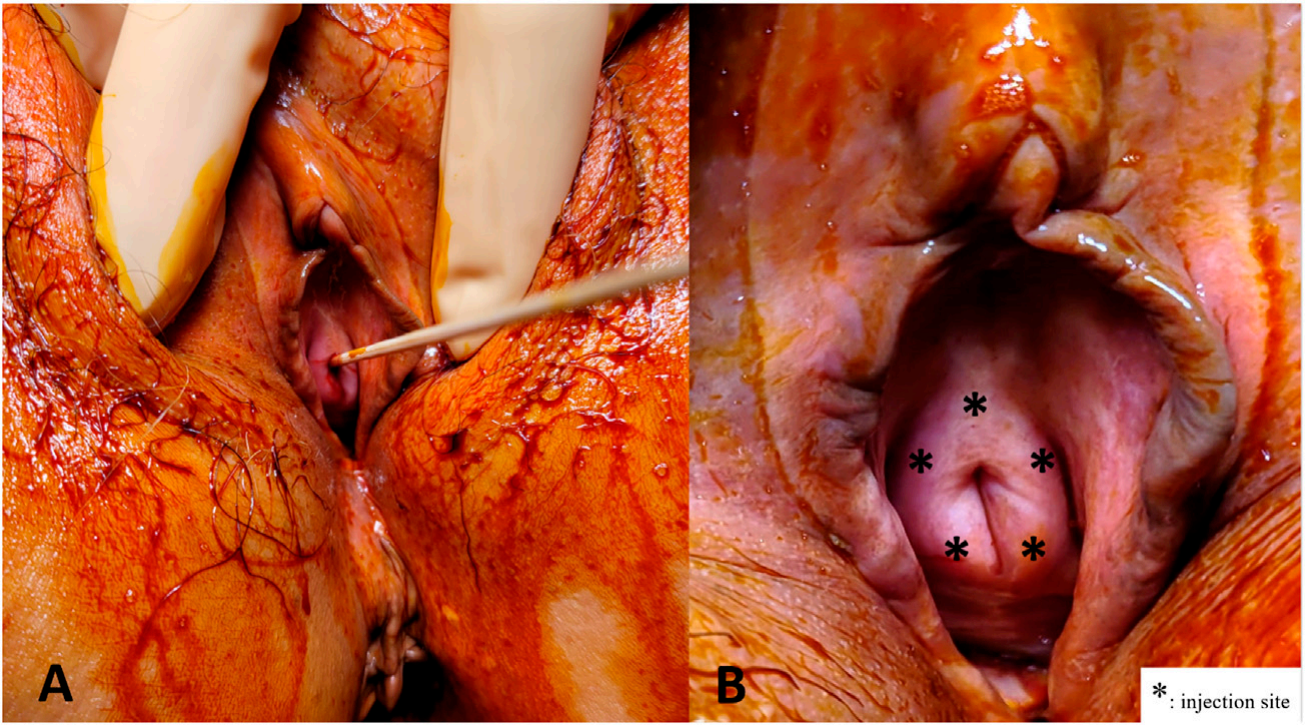
Platelet-rich plasma (PRP) injections for the urethral sphincter. **(A)** A sterile 6" cotton tipped applicator was inserted to determine the axis of urethra. **(B)** The PRP injections were administered at five sites (*) from the perineum around the urethral meatus in female patients.

All subjects received the urethral sphincter injection under intravenous general anesthesia in the operating room. The urethral sphincter PRP injections were administered at 5 sites around the urethral meatus (2, 5, 7, 10, and 12 o’ clock positions) (Figure 1). One ml of PRP was injected at each site. Patients did not receive urethra catheterization after the procedure and were discharged on the same day if no complication developed. Possible complications related to injection, such as hematuria, micturition pain, difficulty urinating, transient urinary retention, and urinary tract infection were informed before treatment. The procedure was repeated every 1 month for a total of 4 treatments within 3 months. The patient completed the outpatient clinic follow-up every 1 month after each urethral injection of PRP, and 3, 6, 9, 12 months after the fourth PRP treatment. Repeated VUDS and ALPP studies were performed at 3 months after the fourth PRP injection to evaluate the changes in the lower urinary tract functions.

The primary treatment outcomes were assessed by the GRA score, categorized from −3, −2, −1, 0, 1, 2, and 3, indicating markedly worsen (−3) to markedly improved status (+3) ((Sant et al., 2003)), reported by the patients themselves after PRP treatment. The primary endpoint of this study was the GRA score after four PRP treatments. A GRA score of ≥ 2 was considered indicative of treatment success. The secondary endpoints included the dry rate after PRP treatments, changes of SUI VAS, UDI-6, IIQ-7 and urodynamic parameters from baseline to 3 months after the fourth PRP treatment, and VAS followed up to 12 months after the fourth PRP treatment.

The categorical variables were presented as frequency (proportion), and the continuous variables were presented as mean ± standard deviation. The Wilcoxon’s signed-rank test was used to distinguish the difference of variables in patients between baseline and after treatment. Wilcoxon’s rank sum test was used for statistical comparisons of variables in between-subgroup analysis. A *p*-value of <0.05 was considered to indicate statistical significance. All statistical analyses were performed on a personal computer using the Statistical Package for the Social Sciences software for Windows (version 25.0; SPSS Chicago, IL, United States).

## 3 Results

A total of 26 women with a mean age of 61.7 ± 15.3 (range 20–88) years and a mean SUI duration of 40.7 ± 22.5 (range 13–178) months were enrolled in this study. Among the enrolled women, 12 (46.2%) presented with a VAS of 1–5, 9 (34.6%) with a VAS of 6–9, and 5 (19.2%) with a VAS of 10. All patients needed pad protection before the PRP treatment. The mean follow-up time after the entire treatment course was 12.6 ± 7.9 months (range, 12–26 months).

The post-treatment mean GRA was 1.5 ± 1.1 (range: 0–3, Table 2). After 4 PRP treatments, 21 (80.8%) women reported a positive response in alleviating SUI, including 13 (50%) achieved a successful outcome (GRA ≥2) and 8 (30.8%) experienced mild improvement (GRA = 1). The success rate was higher in procedure-naïve patients (6 of 14, 58.3%) than the patient who had previous sling surgery (7 of 12, 42.9%), however, the GRA between these two subgroups did not reach statistical significance (1.6 ± 1.3 vs. 1.5 ± 0.9, *p* = 0.695).

**TABLE 2:** Clinical outcomes and change of urodynamic parameters after platelet-rich plasma urethral sphincter injection treatment.

Variable	Baseline	After treatment	*p* Value
Primary endpoints			
GRA score		Total (*n* = 28)	
GRA score = 0		5 (19.2%)	
GRA score = 1		8 (30.8%)	
GRA score = 2		7 (26.9%)	
GRA score = 3		6 (23.1%)	
Average		1.5 ± 1.1	
Successful outcome (GRA≥2)		13 (50.0%)	
Secondary outcome			
UDI-6	5.1 ± 2.3	3.2 ± 2.5	<0.001
IIQ-7	7.3 ± 4.8	3.9 ± 3.8	<0.001
VAS of SUI	6.4 ± 2.3	3.9 ± 3.1	<0.001
VUDS parameter			
CBC (ml)	360.9 ± 172.2	349.2 ± 125.3	0.767
Vol (ml)	251.5 ± 189.3	269.8 ± 169.5	0.668
PVR (ml)	109.4 ± 161.8	79.4 ± 114.6	0.219
Qmax (ml/s)	10.7 ± 6.7	12.5 ± 7.0	0.248
cQmax (Qmax/voided volume^1/2^)	0.60 ± 0.35	0.66 ± 0.32	0.464
Pdet(cmH_2_O)	12.8 ± 14.0	13.2 ± 11.2	0.857
ALPP (cmH_2_O)	109.9 ± 49.1	155.6 ± 51.0	<0.001
MUCP(cmH_2_O)	39.8 ± 16.8	38.8 ± 13.9	0.639
FPL(mm)	31.5 ± 6.1	31.3 ± 4.8	0.816

Values are presented as mean ± standard deviation or number (%). GRA, global response assessment; UDI-6, urogenital distress inventory; IIQ-7, incontinence impact questionnaire; VAS of SUI, visual analogue scale of stress urinary incontinence severity; VUDS, videourodynamic study; CBC, cystometric bladder capacity; Vol, voided volume; PVR, post-void residual volume; Qmax, maximum flow rate; cQmax, corrected Qmax (Qmax/voided volume^1/2^), Pdet, voiding detrusor pressure at Qmax; ALPP, abdominal leak point pressure; MUCP, maximum urethral closing pressure; FPL, functional profile length.

On the other hand, 5 women (19.2%) had no any improvement of SUI. Among them, 2 had mixed urinary incontinence, 1 was lumbar spinal cord injury, 1 had myasthenia gravis, and 1 had received radical hysterectomy due to cervical cancer. Three of these women converted to a suburethral sling procedure after the failure PRP treatment.

At 3 months after the fourth PRP treatment, 12 (46.2%) patients achieved complete continence and pad-free. At the end of follow-up, 7 (26.9%) patients still remained totally continent without pad use, 14 (53.8%) remained mild SUI with acceptable outcome (3 requested for the extra PRP injection), and the other 2 (7.7%) had SUI relapse and seek for other intervention. Patient with dry outcome was associated with a better GRA treatment outcome (Pearson correlation coefficient = 0.221), but there was no significant correlation between them (*p* = 0.279).

**FIGURE 2:**
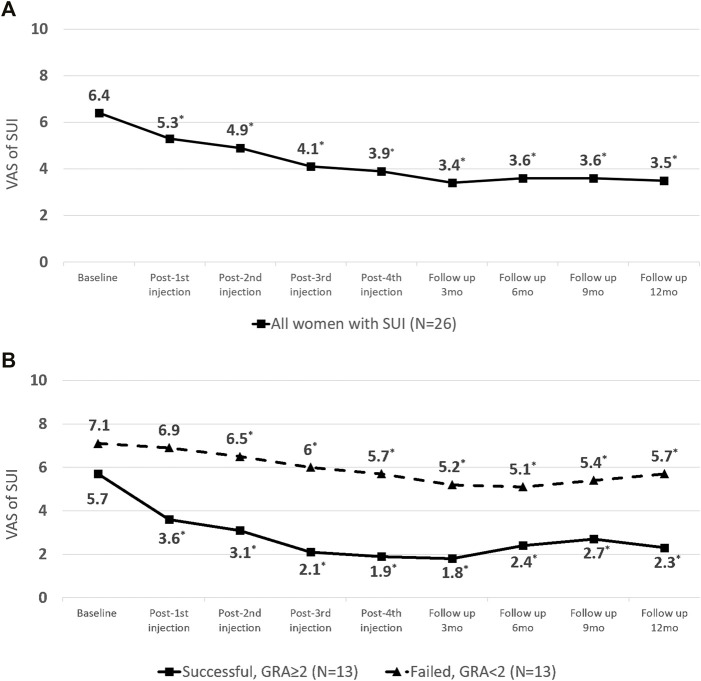
Changes in visual analogue scale (VAS) score of stress urinary incontinence (SUI) from baseline to the end-point of platelet-rich plasma (PRP) treatment **(A)** in all patients. **(B)** Subgroup analysis by outcome.

The severity of SUI reduced significantly at primary end-point (VAS of SUI from 6.4 ± 2.3 to 3.9 ± 2.3, *p* < 0.001) (Table 2; Figure 2), as well as in UDI-6 (5.1 ± 2.3 to 3.2 ± 2.5, *p* < 0.001) and IIQ-7 (7.3 ± 4.8 to 3.9 ± 3.8, *p* < 0.001). During the treatment course, significant therapeutic effects were observed immediately after the first PRP injection and consolidated by the following repeated injections (Figure 2A). Both successful and non-successful subgroups had achieved the similarly prominent results, which was better in those with a success result. The changes in the VAS of SUI after treatment were significantly greater after first PRP injection in successful group and after second PRP injection in non-successful group (*p* = 0.010 and 0.012, respectively; Figure 2B). The therapeutic effect persisted throughout the 1-year period on (average GRA at 12 months was 1.6 ± 1.1), without significant rebounding of average VAS (Figure 2), while 5 patients claimed the recurrence of VAS (≥2 points increase) during the follow-up period. The other VUDS parameters showed no significant difference between baseline and after PRP treatment, with the exception of ALPP, which was significantly increased (109.9 ± 49.1 to 155.6 ± 51.0 cmH_2_O, *p* < 0.001) (Table 2).

Table 3 showed the comparison of baseline characteristics and urodynamic variables between patients with successful and failed treatment outcomes. The results revealed that all potential predictive factors such as the age, medical comorbidities such as diabetes or cerebrovascular accident event, parity history of normal spontaneous delivery, pelvic surgery history or suburethral sling procedure, SUI duration, initial severity of SUI, and lower urinary tract function did not significantly impact the patient’s successful outcomes.

**TABLE 3:** Comparison of the baseline demographics and urodynamic parameters between patients with successful and failed treatment outcomes.

Variable	Success (*n* = 13)	Failed (*n* = 13)	*p* Value
Baseline characteristics			
Age (yr)	65.0 ± 10.2	58.4 ± 19.0	0.274
Diabetes mellitus	2 (15.4%)	3 (23.1%)	0.619
History of CVA event	3 (23.1%)	0 (0%)	0.220
History of NSD	8 (61.5%)	5 (38.5%)	0.434
History of pelvic surgery	2 (15.4%)	2 (15.4%)	1.000
Previous sling procedure for SUI	6 (42.9%)	8 (57.1%)	0.695
SUI duration (months)	37.9 ± 20.1	43.5 ± 24.9	0.424
Baseline VAS of SUI	5.7 ± 1.7	7.1 ± 2.6	0.126
Ave. PLT Con. of PRP (x10^3^/ul)	949.3 ± 433.9	1023.1 ± 388.3	0.658
Ave. PLT multiple of PRP (fold)	4.0 ± 1.4	3.6 ± 1.2	0.510
Baseline VUDS and UPP parameters			
DO	3 (23.1)%	4 (30.8%)	0.658
DU/DODU	5 (38.5%)	3 (23.1%)	0.673
CBC (ml)	444.5 ± 225.1	331.9 ± 151.5	0.148
Vol (ml)	274.5 ± 207.6	217.2 ± 118.3	0.399
PVR (ml)	170.0 ± 270.7	114.6 ± 176.2	0.542
Qmax (ml/s)	11.5 ± 7.2	10.2 ± 5.4	0.628
cQmax (Qmax/voided volume^1/2^)	0.59 ± 0.38	0.61 ± 0.35	0.847
Pdet(cmH_2_O)	12.6 ± 9.7	12.7 ± 15.5	0.992
ALPP (cmH_2_O)	95.8 ± 48.8	124.1 ± 46.9	0.145
MUCP(cmH_2_O)	40.7 ± 16.1	45.8 ± 15.8	0.670
FPL(mm)	30.2 ± 6.0	31.9 ± 3.8	0.451

Values are presented as mean ± standard deviation or number (%). NSD, normal spontaneous delivery; CVA, cerebrovascular accident; DO, detrusor overactivity; DU, detrusor underactivity; GRA, global response assessment; UDI-6, urogenital distress inventory; IIQ-7, incontinence impact questionnaire; VAS of SUI, visual analogue scale of stress urinary incontinence severity; VUDS, videourodynamic study; CBC, cystometric bladder capacity; Vol, voided volume PVR, post-void residual volume; Qmax: maximum flow rate; cQmax, corrected Qmax (Qmax/voided volume^1/2^), Pdet, voiding detrusor pressure at Qmax; ALPP, abdominal leak point pressure; MUCP, maximum urethral closing pressure; FPL, functional profile length.

Table 4 showed the difference in subjective score and urodynamic parameters before and after PRP injections between successful and failed subgroup. Patient with success outcome (GRA ≥2) had significant improvement in the reduction of UDI-6, IIQ-7 and VAS scores after the PRP treatment, but only VAS improvement was observed in the failed subgroup. A significantly greater reduction of VAS was also noted in the successful subgroup (*p* < 0.001). As for VUDS parameters, ALPP showed significant improvement only in successful group (*p* < 0.001) but not in failed cases (*p* = 0.168).

**TABLE 4:** Comparison of the difference in subjective scores and urodynamic parameters before and after PRP treatment between the successful and failed subgroups.

Variable		Success (*n* = 13)	Fail (*n* = 13)	*p* value of Δ (S vs F)
Subjective assessment				
UDI-6	Baseline	4.7 ± 2.3	5.5 ± 2.3	0.061
	Post-OP	1.9 ± 1.1^a^	4.4 ± 2.8	
IIQ-7	Baseline	5.7 ± 4.3	8.7 ± 4.9	0.394
	Post-OP	1.8 ± 2.5^a^	5.8 ± 3.8^a^	
VAS of SUI	Baseline	5.7 ± 1.7	7.1 ± 2.6	<0.001^b^
	Post-OP	1.9 ± 1.9^a^	5.7 ± 2.9^a^	
VUDS and UPP parameters				
CBC (ml)	Baseline	444.5 ± 225.1	331.9 ± 151.5	0.572
	Post-OP	395 ± 134.8	283.7 ± 78.3	
Vol (ml)	Baseline	274.5 ± 207.6	217.2 ± 118.3	0.930
	Post-OP	315 ± 178.5	205.1 ± 143.1	
PVR (ml)	Baseline	170.0 ± 270.7	114.6 ± 176.2	0.433
	Post-OP	80.0 ± 120.6	78.6 ± 115.0	
Qmax (ml/s)	Baseline	11.5 ± 7.2	10.2 ± 5.4	0.464
	Post-OP	14.3 ± 7.1	9.9 ± 6.5	
cQmax (Qmax/voided volume^1/2^)	Baseline	0.59 ± 0.38	0.61 ± 0.35	0.382
	Post-OP	0.71 ± 0.29	0.58 ± 0.36	
Pdet(cmH_2_O)	Baseline	12.6 ± 9.7	12.7 ± 15.5	0.281
	Post-OP	14.2 ± 10.9	11.7 ± 12.4	
ALPP (cmH_2_O)	Baseline	95.8 ± 48.8	124.1 ± 46.9	0.072
	Post-OP	161.4 ± 55.8^a^	149.9 ± 47.2	
MUCP(cmH_2_O)	Baseline	40.7 ± 16.1	43.8 ± 15.8	0.813
	Post-OP	41.8 ± 14.0	49.8 ± 7.4	
FPL(mm)	Baseline	30.2 ± 6.0	31.9 ± 3.8	0.908
	Post-OP	31.1 ± 5.5	32.0 ± 1.4	

a Compared with baseline in groups, *p* value < 0.05;

b Compared between groups, *p* value < 0.05.

Values are presented as mean ± standard deviation or number (%). UDI-6, urogenital distress inventory; IIQ-7, incontinence impact questionnaire; VAS of SUI, visual analogue scale of stress urinary incontinence severity; VUDS, videourodynamic study; CBC, cystometric bladder capacity; Vol, voided volume PVR, post-void residual volume; Qmax, maximum flow rate; cQmax, corrected Qmax (Qmax/voided volume^1/2^), Pdet, voiding detrusor pressure at Qmax; ALPP, abdominal leak point pressure; MUCP, maximum urethral closing pressure; FPL, functional profile length.

During the perioperative course and further entire follow-up period in the cohort, only 1 (3.8%) patient reported straining o urination after PRP injection, the symptom resolved after intermittent self-catheterization for several days. No other remarkable adverse events or complications such as hematuria, micturition pain, acute urinary retention or urinary tract infection developed in further entire follow-up period in this cohort. None of the participants experienced deterioration in terms of the SUI severity after urethral sphincter PRP injection.

## 4 Discussion

This study demonstrated that repeated PRP urethral sphincter injections are effective and safe in alleviating SUI in women. In this study, we focused on female SUI patients with a larger case number and longer follow-up period (up to 1 year) to determine the midterm outcome. After 4 repeated PRP urethral injections, 21 (80.8%) women reported a positive response to the procedure and decrease of the severity in subjective symptom score. A total of 13 (50%) women had a successful outcome, 12 (46.2%) were totally dry at 3 months, and 7 (26.9%) remained continence at 12 months after the PRP treatment.

Platelet-derived products have been widely used in tissue regeneration and recovery, including skeletomuscular defects, skin ulcer or burn, and dental implants (Liggett et al., 1995; Anitua, 1999; Burnouf et al., 2016). It could decrease tissue inflammation and improve urothelial barrier function in IC/BPS patient (Burnouf et al., 2016), and improved SUI severity in patient with postprostatectomy urinary incontinence and non-neurogenic ISD (Jiang et al., 2021; Lee et al., 2021). In this study, the effective results in improving severity of SUI and increase of ALPP, suggesting the urethral sphincter regeneration might be improved and this minimally invasive procedure might have a certain role in the future treatment of female SUI.

Urodynamic evidence in this study showed significantly improved of posttreatment urethral resistance on ALPP in VUDS. The question whether a temporary bulking effect or true regeneration promotion have been raised (Lee et al., 2001). In this study, the clinical response appeared until weeks after the injection, and the therapeutic efficacy could last even up to 1 year after the fourth PRP injection, and 7 (26.9%) patients remained total continence without pad use at the end of 1 year follow-up. In addition, the negative effect on lower urinary tract function such as bladder outlet obstruction or urinary tract infection did not occur. These results all suggest that the efficacy of urethral PRP injection is less likely due to a bulking effect. Nevertheless, a longer follow-up period and investigation is still necessary to ensure true PRP effects and the therapeutic mechanism.

The mechanism of female SUI is multifactorial (Kuo et al., 1994). In addition to the extrinsic factors such as hypermobility of the bladder neck and urethra or concomitant pelvic organ prolapse, the failure of intrinsic continence mechanism was also important to contribute the development of SUI (Harding and Thorpe, 2010). The intrinsic sphincter deficiency involved not only the atrophic urethral mucosa but also submucosal vasculature related connective tissue, smooth muscle and striated sphincter (Haab et al., 1996). Theoretically, cell therapy is plausible that the deficient urethral sphincter may regain its innervation or striated muscle cells. (Williams et al., 2016) In this study, all patients who had a diagnosis of ISD proven by VUDS had a positive response to PRP treatment, suggesting the urethral sphincter deficiency had been adequately treated through repeat PRP injections.

In the five patients who had no response to PRP treatment, several remarkable confounding factors were noted, including mixed urinary incontinence, history of lumbar spinal cord injury, myasthenia gravis, and previous radical hysterectomy. These findings are compatible with the regenerative effects of PRP, which depends on the release of growth factors from the alpha-granules of platelets as well as on the response of the progenitor cells in the damaged tissue (Mussano et al., 2016). Patient with etiology of ISD, such as neurogenic causes, might have less favorable response to urethral PRP treatment. In this proof-f-concept clinical trial, we intended to include these SUI patients because we wanted to demonstrate that PRP has no effect on the tissue with neurogenic deficit.

Urethral bulking agent injection (UBAs) has been widely used as one of the choice in the second-line therapy to treat female SUI after the conservative treatment has failed (Abrams et al., 2018). Acting as a minimally invasive procedure, the cost, efficacy and durability of UBAs are varied and depend on different agent. The short term efficacy of UBAs are generally encouraging, with patient-reported cure rate of 68% at 12 months (Kirchin et al., 2017), and 67.1% of patients with moderate improvement in those encounter prior failed sling procedure (Dray et al., 2019). However, reduced success rates in longer follow-up period of UBAs, and the complications to safety concern have been reported (Hussain and Bray, 2019). In this study, the therapeutic effect of PRP remains up to 1 year, suggesting the therapeutic mechanism should be more than the bulking effect. Urethral PRP injections might have a regenerative effect rather than bulking effect on the increase of urethral resistance. The study revealed an acceptably effective and durable clinical response and less safety concern in PRP treatment. The balance between advantages and disadvantaged of urethral PRP injections seem outweigh that of UBAs.

Several predictive factors that might influence the clinical efficacy of PRP treatment including age and different PRP concentration have been reported. However, it remains controversial depending on different disease nature (Gentile and Garcovich, 2020; Alessio-Mazzola et al., 2021; Shen et al., 2021). In 60 cases of symptomatic knee osteoarthritis receiving homologous intra-articular PRP injections, a less favorable clinical outcome was reported in patients aged ≥ 80 years versus those age 65–79 years (Bottegoni et al., 2016). In this study, the influence of age on clinical outcomes of urethral PRP treatment was not significant. Most of patients in this trial had clinical improvement of SUI severity regardless of the baseline characteristics, suggesting the urethral regenerative ability is present in most women with SUI, except those with neurogenic ISD.

In this study we also noted that the success rate was lower in patients who had recurrent SUI after previous suburethral sling procedure than that in patients of treatment naïve (42.9% vs. 58.3%). Although the difference was not significant, it is likely that the women with previous anti-incontinence surgery might have reduced urethral sphincter muscle volume with ageing and this factor might result in their SUI recurrence and less therapeutic effect of PRP treatment. Nevertheless, since some women still have successful outcome, urethral PRP injections can still be considered as a salvage procedure after previous sling procedure failure.

The other secondary outcome parameter, UDI-6 and IIQ-7, have been used to assess symptom distress and the impact on daily life of urinary incontinence. (Utomo et al., 2015) The result of study suggested a positive response in UDI-6 and IIQ-7 improvement after PRP injections in either successful or failed subgroups. Acting as a less invasive therapy, PRP injection might help to rehabilitate several aspects related to life quality, such as daily workout, exercising, entertainment, social activities, as well as better mood health.

There are some limitations in this study. First, due to ethical consideration, a placebo control arm was lacking with relative small sample size. However, the results of this study provide evidence for a successful outcome at 12-month follow-up, suggesting a placebo effect is not present. A head-to-head comparative study to compare urethral PRP injections with other anti-incontinence procedure may be feasible to determine the definite therapeutic efficacy and durability is needed. Second, the study only provided indirect evidence such as subjective symptomatic improvement and increase of ALPP in urodynamic examinations. ALPP might not correlate with the severity of incontinence and QoL. However, it can be an objective evidence to proof the therapeutic efficacy of PRP treatment on SUI. Therefore, we also survey measured parameters other than ALPP, such as modified VAS and GRA to comprehensively assess therapeutic outcome of the severity of SUI and QoL. Direct evidence such as image of the urethral sphincter volume or histological cell proliferation is lacking to demonstrate the cell regeneration and increased innervation after PRP injections. Third, the standard dose and the optimal preparation of PRP, the injection techniques, and the ideal regimen of repeated PRP injections have not been established. However, the results of this study have suggested the safety and efficacy of urethral PRP injection for SUI in women.

## 5 Conclusion

The study demonstrates the efficacy and safety of repeated urethral sphincter injections of autologous PRP. Repeat PRP urethral injections can increase urethral sphincter resistance and decrease the severity of SUI in women with the therapeutic effect lasting up to 12 months in most of patients who responded to PRP treatment, with acceptable adverse events.

## Data Availability

The original contributions presented in the study are included in the article/Supplementary Material, further inquiries can be directed to the corresponding authors.
