# Supplementary material for: The Efficacy and Mid-term Durability of Urethral Sphincter Injections of Platelet-Rich Plasma in Treatment of Female Stress Urinary Incontinence
Source: Front Pharmacol. 2022 Feb 8;13:847520. doi: 10.3389/fphar.2022.847520 (PMC8861290; doi:10.3389/fphar.2022.847520)
Supplement: Supplementary file 1 [file DataSheet1.docx]

**Supplementary 1. Inclusion and exclusion criteria**

*Inclusion Criteria*

Patients who qualified all the following criteria were eligible for inclusion:

1. Age ≥20 years
2. Patients with predominant symptom of stress urinary incontinence for more than one year
3. Proven to have intrinsic sphincter deficiency by video-urodynamic study in the preceding 1 year
4. Patients who had previously received conservative treatment or anti-incontinence surgery but still had mild stress urinary incontinence
5. Provision of written informed consent by the patient or his/her legally acceptable representative

*Exclusion Criteria*

1. Patients with severe disease of heart, lung, liver or kidney
2. Patient with active urinary tract infection
3. Patient with severe uncorrected pelvic organ prolapse.
4. Patient with known bladder outlet obstruction at enrollment.
5. Patients not able to receive regular follow-up
6. Patients with any contraindication for urethral catheterization during treatment
7. Patient in pregnant and lactating, or child-bearing potential without contraception.
8. Patients with critical thrombocytopenia, anticoagulant use or hypofibrinogenemia
9. Patients who participated in other investigational drug trials within 3 months before enrollment
10. Patients unable to record 3-day voiding diary
